# In SARS-CoV-2, astrocytes are in it for the long haul

**DOI:** 10.1073/pnas.2209130119

**Published:** 2022-07-18

**Authors:** Shuhan Huang, Gord Fishell

**Affiliations:** ^a^Department of Neurobiology, Blavatnik Institute, Harvard Medical School, Boston, MA 02115;; ^b^Stanley Center for Psychiatric Research, Broad Institute of MIT and Harvard, Cambridge, MA 02142;; ^c^Program in Neuroscience, Harvard Medical School, Boston, MA 02115

The most dire consequences of severe acute respiratory syndrome coronavirus 2 (SARS-CoV-2) infection may not manifest during the initial infection but appear later and are disproportionately associated with compromised neural function. Central nervous system (CNS) complications often appear after the acute phase but persist and become prominent later and last for weeks to months (i.e., long COVID). Indeed, while severe neurological diseases accompanying COVID-19 are rare during the acute phase (1, 2), 7.3 to 33.62% of COVID-19 survivors experience long COVID (3, 4), with diverse neurological and neuropsychiatric complications such as headaches, fatigue, anosmia, cognitive impairment, and depression. In addition, surprising brain pathological changes, such as loss of gray matter in several regions of cortex, have been identified in COVID-19 survivors (5). With hundreds of millions of people being affected by SARS-CoV-2 worldwide, concerns have been raised in regard to public health and socioeconomic burdens. One of the key questions remains whether SARS-CoV-2 can infect brain cells directly. To address this, Andrews et al. (6) investigated the primary root of infection in the CNS and presented evidence that SARS-CoV-2 preferentially infects astrocytes in the brain through noncanonical mechanisms. This indicates that the neurological symptoms associated with long COVID arise indirectly from increased neuroinflammation and nonautonomous neuronal death.

**Fig. 1.**
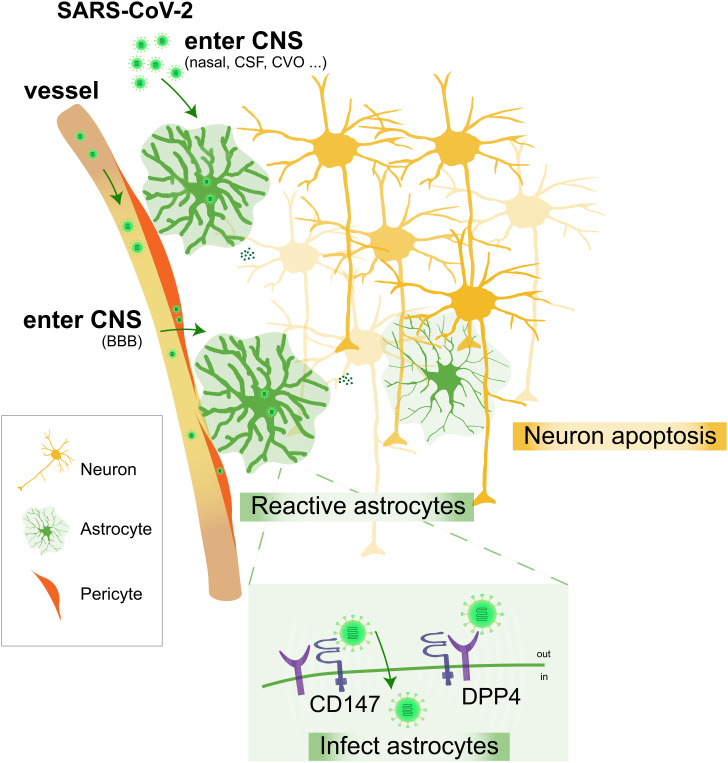
Astrocytes are the primary targets of SARS-CoV-2 in the brain. SARS-CoV-2 is shown to preferentially infect astrocytes over neurons in primary and organoid cortical cultures, resulting in astrocyte reactivation and non-cell-autonomous neuronal death. Furthermore, BSG/CD147 and DPP4 are found to be key molecular mediators of SARS-CoV-2 infection in cortical astrocytes. Abbreviations: BBB, blood–brain barrier; CSF, cerebrospinal fluid; CVO, circumventricular organs.

Foremost to understanding SARS-CoV-2’s impact on the CNS is distinguishing which cells are impacted by this virus and whether these effects are direct or indirect. Given the complexity and diversity of the CNS symptoms, discerning whether health impacts arise from direct infection of the nervous system or indirect hypoxia, coagulopathy, and systemic inflammation that are able to trigger downstream neuroinflammatory responses is essential. Central to addressing these issues is determining which CNS populations are directly infected by the virus. To date, inferences of SARS-CoV-2 infection have largely come from examination of postmortem tissue. Neurotropism, referring to the ability of a virus to infect and replicate in neural tissue, of SARS-CoV-2 remains unclear. Recently, groups have utilized human pluripotent stem cell (hPSC)-based models as a convenient tool to investigate neurotropism of SARS-CoV-2 on various brain cell types and perform large-scale screening of therapeutic targets (7). Interestingly, a study using neural-perivascular organoids identified pericytes in the vascular unit potentially mediate the entry of SARS-CoV-2 into the brain and the spread to astrocytes (8), whose endfeet are in contact with pericytes. In PNAS, Andrews et al. (6) further explored the neurotropism of SARS-CoV-2 using hPSC-derived cortical organoids, as well as primary human cortical tissues during development and in adulthood (Fig. 1). Bolstering the earlier report, they found that SARS-CoV-2 preferentially infects and replicates and propagates in astrocytes, particularly those adjacent to infected vasculature. Notably, this vulnerability of astrocytes to SARS-CoV-2 not only exists during development but also extends to adulthood. In contrast, neurons and microglia are less likely to be directly infected. Importantly, while microglia and astrocytes are both reactivated, a direct dosage-sensitive effect of SARS-CoV-2 is only observed in reactive astrocytes.

Many studies have suggested that SARS-CoV-2 relies on its obligate receptor to enter cells (9). The canonical SARS-CoV-2 receptor, angiotensin-converting enzyme 2 (ACE2), is the major entry receptor for SARS-CoV-2 in many cell types, such as nasal epithelial cells, endothelial cells, and pericytes (10). However, ACE2 expression is undetectable in cortical astrocytes pre- or postinfection. If not ACE2, what are the molecular mechanisms mediating SARS-CoV-2 infection on cortical astrocytes? Andrews et al. (6) first investigated the expression of neuropilin 1 (NRP1), a host factor that can enhance transmembrane serine protease 2 (TMPRSS2)-mediated entry of wild-type SARS-CoV-2 (11). However, NRP1, like ACE2, was not detected in cortical cells that are infected. The receptor basigin (BSG/CD147), which is abundantly coexpressed with SARS-CoV-2 proteases furin (FURIN) and cathepsin B (CTSB) in pericytes and astrocytes, represents an alternative route for SARS-CoV-2 entry (12). In addition, dipeptidyl peptidase 4 (DPP4), the main receptor of Middle East respiratory syndrome–related coronavirus, has been suggested as a binding target for SARS-CoV-2 (13). In PNAS, Andrews et al. (6) demonstrate that DPP4 and BSG/CD147 are able to mediate SARS-CoV-2 infection in astrocytes. Specifically, while knockdown of BSG/CD147 or treatment with DPP4 inhibitor (vildagliptin) significantly reduces SARS-CoV-2 infection, conversely overexpressing BSG/CD147 or DPP4 increases infection in vitro. Both double-stranded RNA (dsRNA)+ cells and N+ cells are increased with DPP4 overexpression, while only dsRNA+ cells are increased with BSG/CD147 overexpression. Taken together, these results suggest that the molecular mechanisms underlying the neurotropism of SARS-CoV-2 are likely mediated by DPP4 and BSG/CD147, which enhances SARS-CoV-2 entry and is important for replication, respectively.

What are the functional consequences of SARS-CoV-2 infection of cortical astrocytes? First of all, Andrews et al. (6) found that infected astrocytes show increased reactivity and cellular stress. Moreover, non-cell-autonomous inflammatory effects are found in SARS-CoV-2–infected cultures, such as an increase in reactive microglia and an overall loss of neurons by apoptosis. Studies have suggested that astrocytes are critical support cells in the regulation of brain energy, metabolism, and microenvironment (14). Interestingly, BSG/CD147 is also a key part of the astrocyte metabolic pathways, providing energy support to neurons (15). Therefore, SARS-CoV-2 infection in astrocytes may cause neuronal death indirectly through inflammation and dysfunction of brain energy metabolism.

To summarize, Andrews et al. (6) found that SARS-CoV-2 can infect brain astrocytes via DPP4 and BSG/CD147, resulting in elevated inflammation and neuronal death. Although very few published autopsy studies have reported detection of SARS-CoV-2 infection in the brain, authors of a preprint from Brazil who analyzed 26 postmortem brains from individuals who died with COVID-19 found that 5 of them had genetic viral components, as well as SARS-CoV-2 spike protein in the brain (16). Moreover, the majority of these SARS-CoV-2 spike+ cells (65.93%) were astrocytes (16), suggesting astrocytes are the main target cell type in the brain. In circumventing the brain’s immune-privileged status, possible neuroinvasive routes for SARS-CoV-2 include the olfactory system, cranial nerves, dysfunctional blood–brain/cerebrospinal fluid barrier, and circumventricular organs (10, 17). In infecting astrocytes, it is likely that the virus circulates in the brain vasculature and infects pericytes, which in turn is spread into astrocytes through their endfeet. Astrocytic infection results in dysfunction of their metabolic homeostasis, enhancing neuroinflammation and impacting energy support for neurons indirectly. These could contribute to COVID-19–associated CNS complications. More severe neurological and neuropsychiatric symptoms may result from neuronal death or synaptic loss in brain regions that are more vulnerable to pathogenesis, inflammation, or energy deficiency. Since the CNS complications are diverse within different individuals and viral infection is a dynamic process, it will be important to further investigate the correlation between viral load within different brain regions, the neuroimmune response levels, and the CNS symptoms with a larger clinical sample size. Furthermore, it will also be crucial to compare different SARS-CoV-2 variants with regard to their ability to infect brain cell types and understand their correlations to CNS symptoms.

If SARS-CoV-2 infection of brain astrocytes in COVID-19 patients is the proximal cause of the observed neurological dysfunctions, the question arises as to how to prevent viral entry preinfection, as well as the means to relieve the ensuing CNS symptoms postinfection. Possible strategies may include, but are not limited to, the blockage of viral infection or replication in pericytes or astrocytes and interventions to reduce neuroinflammation. To relieve the symptoms, approaches to augment metabolic energy to the brain as a means to restore brain homeostasis may also have promise. Although brain organoids do not yet fully replicate the complete cellular and regional diversity in the brain, they are a powerful tool to understand the mechanism of viral tropism and screen for treatment targets. Together, the combination of clinical observations, the in vitro use of human tissues, and organoid cultures will help us better understand COVID-19 pathology in humans and facilitate the development of treatments against SARS-CoV-2 infection.
